# 

**Published:** 1845-03

**Authors:** 


					﻿THE
WESTERN JOURNAL
OF
MEDICINE AND SURGERY:
EDITED BY
DANIEL DRAKE, M. D.
AND
LUNSFORD P. YANDELL, M. D.
PROFESSORS IN THE LOUISVILLE MEDICAL INSTITUTE,
AND
THOMAS W. COLESCOTT, M. D.
NO. LXIII.—MARCH, 1845.
NEW SERIES.
VOL. III.—NO. III.
LOUISVILLE, KY.
PUBLISHED MONTHLY, BY PRENTICE AND WEISSINGER,
PROPRIETORS AND PRINTERS.
1845.
This Number contain* 4 sheet*—t'osiaoe vtmtT 100 writs (> czzHSj oter 100 n.dts 10 cents
				

